# Innate lymphoid cells, possible interaction with microbiota

**DOI:** 10.1007/s00281-014-0470-4

**Published:** 2014-12-13

**Authors:** Kazuyo Moro, Shigeo Koyasu

**Affiliations:** 1 Laboratory for Immune Cell Systems, RIKEN Center for Integrative Medical Sciences (IMS), 1-7-22 Suehiro-cho, , Tsurumi-ku, Yokohama, 230-0045 Japan; 2 Precursory Research for Embryonic Science and Technology (PRESTO), Japan Science and Technology Agency, 7 Goban-cho, Chiyoda-ku, Tokyo 102-0076 Japan; 3 Division of Immunobiology, Department of Medical Life Science, Graduate School of Medical Life Science, Yokohama City University, 1-7-29 Suehiro-cho, , Tsurumi-ku, Yokohama, 230-0045 Japan; 4 Department of Microbiology and Immunology, Keio University School of Medicine, 35 Shinanomachi, Shinjuku-ku, Tokyo 160-8582 Japan

**Keywords:** ILC, Epithelia, Infection, Homeostasis, Allergy, Inflammation, Plasticity

## Abstract

Recent studies have identified novel lymphocyte subsets named innate lymphoid cells (ILCs) lacking antigen-specific receptors. ILCs are present in a wide variety of epithelial compartments and occupy an intermediate position between acquired immune cells and myeloid cells. ILCs are now classified into three groups: group 1 ILC, group 2 ILC, and group 3 ILC based on their cytokine production patterns that correspond to the helper T cell subsets Th1, Th2, and Th17, respectively. ILCs play important roles in protection against various invading microbes including multicellular parasites, and in the maintenance of homeostasis and repair of epithelial layers. Excessive activation of ILCs, however, leads to various inflammatory disease conditions. ILCs have thus attracted interests of many researchers in the fields of infectious immunity, inflammatory diseases, and allergic diseases. Because epithelial cells sense alterations in environmental cues, it is important to understand the functional interaction between epithelial cells, ILCs, and environmental factors such as commensal microbiota. We discuss in this review developmental pathways of ILCs, their functions, and contribution of commensal microbiota to the differentiation and function of ILCs.

## Immune cells in innate immunity

Immune cells are divided into two categories, acquired immune cells and innate immune cells, based on their ability to recognize specific antigens. T cells and B cells recognize specific antigens by their unique antigen receptors, which are wholly dependent on recombination-activating genes (*Rag1* and *Rag2*) for the acquisition of antigen specificities. Innate immune cells do not have specific antigen receptors and can be divided into two types, myeloid cells and innate lymphoid cells (ILCs). Although there is no clear definition for the myeloid and lymphoid classifications, it is believed that small and round cells with a high nucleus/cytoplasm ratio are lymphoid cells. The development of myeloid cells is dependent on PU.1, a member of the Ets family of transcription factors. PU.1 is not specific to myeloid cell development, however, since B cells are absent in PU.1-deficient mice [1]. Myeloid cells express pattern recognition receptors (PRRs) such as Toll-like receptors (TLRs) and C-type lectin receptors (CLRs) on their cell surface and recognize molecular structures on microbes. The major roles of these cells are phagocytosis to kill invading microbes and antigen processing to present antigens to acquired immune cells. ILCs, which are morphologically similar to T and B cells, occupy an intermediate position between acquired immune cells and myeloid cells. The helix-loop-helix transcription factor inhibitor of DNA binding 2 (Id2) and cytokine signaling through cytokine receptor common gamma chain (γ_c_) are required for the development of all ILCs. While acquired immune cells and myeloid cells work cooperatively, ILCs respond directly to cytokines from both myeloid cells and non-immune cells such as epithelial cells and play crucial roles in the protection of epithelial barriers against infections, repair of epithelial integrity, and maintenance of organ homeostasis.

Natural killer (NK) cells have long been an orphan innate lymphocyte population because they have been considered a member of lymphocytes but lack antigen receptors. In the late 1990s, another type of lymphocyte-lacking antigen receptors was found in fetal tissues. These cells play crucial roles in the differentiation of lymphoid organs such as lymph nodes and Peyer’s patches and were named lymphoid tissue inducer (LTi) cells. During the last several years, new members of the ILC family have been reported from many laboratories and studies on ILCs are on the rise, resulting in reports of various ILCs in many tissues with different proposed names. To clarify the identities of ILCs and classify ILCs based on their functions, a panel of researchers within this field introduced a system for the classification and naming of ILCs and reported their proposal in Nature Reviews Immunology in 2013 [2]. In this review article, ILCs were divided into three groups: group 1 ILC (ILC1 and NK cells), group 2 ILC (ILC2s), and group 3 ILC (ILC3s and LTi) based on their cytokine production patterns that correspond to the helper T cell subsets Th1, Th2, and Th17, respectively. This classification has been generally accepted by researchers in this field, but as developmental pathways and functions of ILC subsets continue to be extensively studied, we faced some incongruities with these descriptions. For example, classical or conventional NK (cNK) cells that are classified as group 1 ILC seem to have distinct developmental pathways to other cell types within this group. Similarly, LTi cells appear to depend on different transcription factors for their differentiation among group 3 ILCs. ILC1, ILC2s, and ILC3s are all dependent on a transcription factor promyelocytic leukemia zinc finger (PLZF), but differentiation of cNK cells and LTi cells is independent of PLZF.

## Common and distinct differentiation pathways of ILCs

**Fig. 1:**
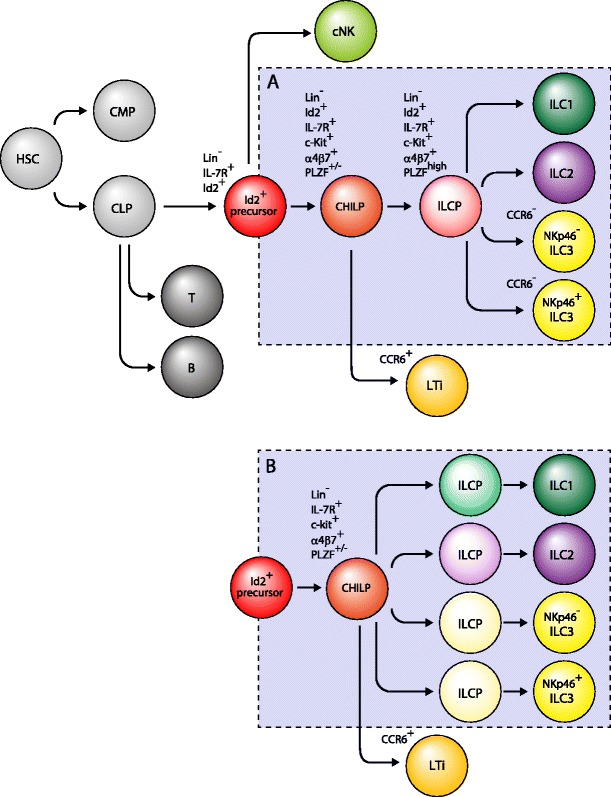
Developmental pathways of ILCs. All ILCs require Id2 signaling for their development. cNK cells branch out earlier than other helper-like ILCs and LTi cells branch out from CHILP. Cell fate determination processes of ILC, ILC2s, and ILC3s from PLZF^+^ ILCP are still unknown, but there are two possibilities: **a** determined after PLZF^+^ ILCP or **b** cell fate determinations are already established before or at the PLZF^+^ ILCP stage

All lymphocytes are derived from common lymphoid progenitors (CLP) in the fetal liver and adult bone marrow and are characterized by the lineage (Lin)^−^c-Kit^int^Sca-1^+^IL-7Rα^+^ phenotype. Knockout studies have identified the transcription factor Id2 and cytokine signals mediated by γ_c_ as essential factors for the development of all ILCs. T and B cells develop independent of Id2 but require *Rag1* and *Rag2* genes for recombination of their antigen receptors. On the other hand, *Rag1* and *Rag2* genes are dispensable for differentiation of ILCs. Interestingly, Yang et al. showed by fate mapping analysis that a fraction of ILC2s once expressed *Rag2*, demonstrating the close relationship between antigen receptor-expressing lymphocytes and ILCs [3]. It was later shown that *Gata3* is critical for the differentiation of ILC1, ILC2s, ILC3s, and LTi but not for cNK cells. Klose et al. recently reported the existence of a Lin^−^Id2^+^IL-7Rα^+^CD25^−^α4β7^+^Flt3^−^ progenitor population that they named common helper-like innate lymphoid cell progenitor (CHILP) capable of developing into all ILC subsets except cytotoxic cNK cells, indicating that cNK cells are distinct from other ILCs [4]. E4BP4 or NF-IL3 was originally reported as an essential transcription factor for cNK cell differentiation, but it was later shown that the lack of E4BP4 impairs the differentiation of all ILCs by the reduction of CHILP, indicating that E4BP4 also controls the differentiation of all ILCs, not only that of cNK cells. In addition, Constantinides et al. found that PLZF, which has been known to control differentiation of innate-type CD1d-restricted NKT cells [5, 6], is transiently expressed in CHILP during ILC differentiation. Fate mapping studies for the expression of *Zbtb16*, the gene encoding PLZF, showed labeling of ILC1, ILC2s, and ILC3s but not cNK cells and LTi cells [7]. Consistent with this observation, *Zbtb16*-deficient mice showed normal development of cNK cells and LTi cells whereas differentiation of ILC1, ILC2s, and ILC3s were variously affected. Accordingly, PLZF^+^ CHILP differentiates to ILC1, ILC2s, and ILC3s but not to cNK cells or LTi cells based on adoptive transfer experiments and clonal differentiation assays [7]. These recent results collectively showed that cNK cells branch out from CLP prior to differentiation of CLP to CHILP, followed by branching out of LTi before acquisition of PLZF expression by CHILP. Lineage specification of ILC1, ILC2s, and ILC3s from PLZF^+^ CHILP takes place by acquisition of T-bet, Gata3, and RORγt expression, respectively (Fig. 1a).

## Group 1 ILC

**Fig. 2:**
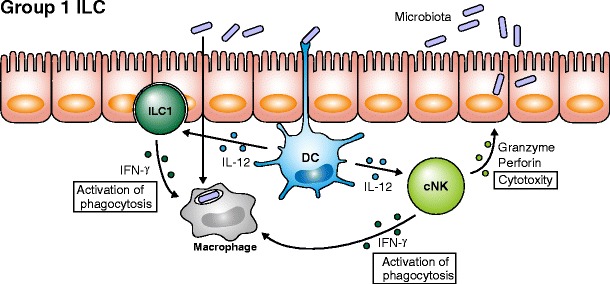
Role of group 1 ILCs. Bacteria captured by dendritic cells (DC) induce IL-12 production to stimulate both conventional NK cells (cNK) and ILC1, both of which produce IFN-γ and accelerate macrophage phagocytosis. cNK cells exhibit cytotoxic activity by degranulating granzyme and perforin and induce apoptosis of target cells such as infected epithelial cells

Studies on group 1 ILC began with the discovery of cNK cells that exhibit cytotoxic activity against tumor cells [8, 9]. Unlike cytotoxic T lymphocytes (CTLs), NK cells lack T cell receptors to recognize complexes of antigen-derived peptides and major histocompatibility complex (MHC). Instead, NK cells utilize CLRs to recognize various molecules expressed on target cells that are mostly induced by cellular stresses such as viral infection. NK cells also recognize self MHC molecules by a unique set of receptors acting as inhibitory receptors to suppress activation signals. Target cells of self-origin can be killed by NK cells when self MHC molecules are downregulated. This can often be observed in tumor cells and in infection by herpes viruses or when self MHC molecules are heavily modified by viral infection. It is also known that NK cells are potent producers of IFNγ upon activation. Furthermore, NK cells produce IFNγ in response to IL-12 derived from myeloid cells such as macrophages and dendritic cells, and IL-18 further enhances IFNγ production by NK cells. NK cells thus have dual functions, namely cytotoxicity and cytokine production (Fig. 2).

NK cells express *Tbx21* (T-bet), critical for IFNγ expression, and secrete granules containing granzyme B and perforin, both of which induce apoptosis of target cells such as cancer cells and cells infected with intracellular microbes. Among γ_c_ cytokines, IL-15 is essential for the differentiation of cNK cells, and unlike other ILCs, IL-7 is dispensable for cNK differentiation [8]. In 2006, DiSanto and colleagues identified thymic NK cells that show less cytotoxic activity than cNK cells but express higher amounts of IFNγ than cNK cells [10]. It was intriguing at that time that differentiation of thymic NK cells was dependent on IL-7 and Gata3 but independent of IL-15, raising the possibility that there are at least two distinct lineages for NK cells. An NK-like population that expresses T-bet and produces IFNγ in response to IL-12 but expresses low levels of granzyme B and perforin was later reported, and this population was termed ILC1 [11]. ILC1 are present in mucosal tissues and share functional features with tissue-resident memory CD8 T cells that require T-bet and E4BP4 for their development and contribute to the pathophysiology of IBD [12]. While cytotoxic cNK cells express perforin, granzyme B, CD56, CD16, CD94, and NKp46, ILC1 are negative for these markers and express, CD161 and CD69, suggesting the presence of at least two phenotypically and functionally distinct populations among group 1 ILCs [11, 12] (Fig. 2).

As mentioned above, Klose et al. recently reported the existence of a Lin^−^Id2^+^IL-7Rα^+^CD25^−^α4β7^+^Flt3^−^ CHILP capable of developing into all ILC subsets except cNK cells, indicating that cytotoxic cNK cells are distinct from other helper-like ILCs [4]. Furthermore, Lin^−^Id2^+^IL-7Rα^+^CD25^−^α4β7^+^Flt3^−^ progenitor cells are able to differentiate into an NKp46^+^IL-7Rα^+^ ILC lineage, which have strong helper function due to IFNγ production and are called ILC1. Both cytotoxic NK cells and ILC1 constitutively express T-bet but differ in the cytokines required for their development. cNK cells depend on IL-15 but not IL-7 [13] while all other ILCs depend on IL-7 but not IL-15. It has been reported that early pre-pro NK cells and immature NK cells express high levels of IL-7Rα [14], but the IL-7 requirement for ILC1 is less well understood. Taken together, these results clearly define two developmentally distinct group 1 ILCs leading researchers within the field to refer to cytotoxic NK cells as cNK cells and to use the term “ILC1” to refer to Lin^−^Id2^+^IL-7Rα^+^CD25^−^α4β7^+^Flt3^−^ derived non-cytotoxic IFNγ-producing cells that have helper functions (Fig. 1). The name “group 1 ILC” is the all-inclusive term for conventional NK cells and ILC1. Moreover, the evidence suggests that the term ILC1 is likely not a suitable abbreviation for “group 1 ILC”.

Microbiota are considered to be a critical factor for lymphoid organogenesis, maintenance of epithelial homeostasis, and development of acquired immune cells. Unlike acquired immune cells, cNK cells do not require commensal bacteria for their development [15]. There are indeed no differences in the expression of KLRG1, CD122, CD49b, NKG2D, and NKp46 on cNK cells between specific-pathogen-free (SPF) and germ-free (GF) housed mice. However, it has been demonstrated that expression of granzyme B and IFNγ were significantly suppressed in GF mice as a result of lack of priming signals that mostly depend on dendritic cells (DCs). Although contribution of commensal bacteria to ILC1 differentiation and function is not well understood, a critical relationship via epithelial cells likely exists between commensal bacteria and ILC1.

## Group 2 ILC

We started our work on group 2 ILC based on our finding of previously unidentified lymphoid clusters in adipose tissue, which we termed fat-associated lymphoid cluster (FALC) [16]. During the process of cellular analysis of FALC, we found a unique population of cells that express c-Kit and Sca-1 but not lineage markers. Up until that point, cells with the Lin^−^c-Kit^+^Sca-1^+^ phenotype were called LSK, a cell population that contains hematopoietic stem cells and progenitor cells capable of differentiating into a variety of immune cell types. However, FALC Lin^−^c-Kit^+^Sca-1^+^ cells did not exhibit any developmental potential to other types of cells either in vivo or in vitro, suggesting that these cells are different from typical LSK cells. High expression of Th2-related genes in FALC Lin^−^c-Kit^+^Sca-1^+^ cells revealed from microarray analysis led us to reconsider the identity of this population: these cells are mature cells but not progenitor cells. A unique characteristic of this population was their ability to constitutively produce type 2 cytokines including IL-5, IL-6, and IL-13. Indeed, FALC Lin^−^c-Kit^+^Sca-1^+^ cells were able to support cell division of B1 B cells in a simple co-culture system, through IL-5 that is constitutively produced by this population. Furthermore, co-cultivation of FALC Lin^−^c-Kit^+^Sca-1^+^ cells with splenic B cells resulted in the production of IgA, demonstrating the helper function of FALC Lin^−^c-Kit^+^Sca-1^+^ cells. Type 2 cytokines are known to be important in anti-helminth immunity, and it was well known that IL-5 and IL-13 are rapidly produced upon helminth infection before a helminth-specific adaptive Th2 response is established, demonstrating the presence of type 2 innate immune responses against helminth infection. We therefore wondered if FALC Lin^−^c-Kit^+^Sca-1^+^ cells are involved in the innate immune response against helminth infection. To demonstrate the role of FALC Lin^−^c-Kit^+^Sca-1^+^ cells in anti-helminth immunity, we focused on two previous reports demonstrating strong type 2 immune responses triggered by IL-25 and IL-33 [17, 18]. We subsequently found that IL-33 and a combination of IL-2 plus IL-25 can induce production of large quantities of type 2 cytokines such as IL-5 and IL-13 in FALC Lin^−^c-Kit^+^Sca-1^+^ cells. Furthermore, we demonstrated that these cells contribute to protection against helminth infection mediated by eosinophilia and goblet cell hyperplasia. Therefore, we termed Lin^−^c-Kit^+^Sca-1^+^ cells natural helper (NH) cells based on their helper functions to both immune and non-immune cells [16].

**Fig. 3:**
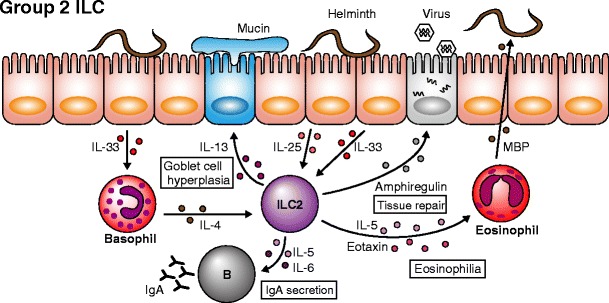
Role of group 2 ILCs. In steady state conditions, ILC2s support the maintenance of IgA secretion from B cells in the intestine and B1 cell survival in the peritoneal cavity. During helminth infection, epithelial cells produce IL-25 and IL-33 to activate ILC2s, and both cytokines induce IL-5, eotaxin and IL-13 production by ILC2s. IL-5 and eotaxin are important for the expansion and migration of eosinophils, and IL-13 induces goblet cell hyperplasia and mucin production. Eosinophils degranulate enzymes such as major basic protein (MBP) to attack the worm, and mucin produced by goblet cells rinses out the worm from the intestinal lumen. IL-33 also stimulates basophils in the early phase of infection to produce IL-4, which induces the initial IL-5 and IL-13 production by ILC2s

A similar cell type, termed nuocytes, was later reported using GFP reporter mice in which GFP was inserted into the coding region of *Il13* gene [19]. Nuocytes were defined as GFP^+^ cells upon administration of IL-25 or IL-33, but a corresponding population in naïve mice was obscure. Nuocytes are slightly different from NH cells because they are induced in the lymph node or spleen following IL-25 injection and express MHC class II but not CD25, unlike NH cells. However, the role of nuocytes is similar to that of NH cells in that they are also important in anti-helminth immunity. It is currently unclear whether NH cells and nuocytes are derived from the same lineage, and the possibility exists that nuocytes are IL-25-activated NH cells. Both cell types are current members of group 2 ILC. It is confusing that while ILC1 is a subset of group 1 ILCs, the term ILC2 is used as an abbreviated term for group 2 ILC. To clarify the distinction between subset name and group name, we recommend that group 2 ILC be called ILC2s. Following reports on the discovery of ILCs, there were a series of reports demonstrating the role of ILC2s in mice and humans [20–31]. ILC2s are localized not only in adipose tissues and lymphoid organs but also in other peripheral tissues including liver, lung, intestine, bone marrow, body cavity, dermis, and peripheral blood [23, 29, 31–35]. ILC2s are involved in anti-helminth innate immunity as well as in other conditions including, but not limited to asthma, atopic dermatitis, cancer, and hepatic fibrosis. Research toward understanding the role of ILC2s in asthma has been extensively performed. Strong type 2 cytokine production by ILC2s that results in eosinophilia and goblet cell hyperplasia is critical for the expulsion of worms during helminth infection (Fig. 3). However, induction of eosinophilia and goblet cell hyperplasia is also known to exacerbate symptoms in various allergic diseases such as asthma. Neutrophil-dominant asthma was once thought to be much more severe than eosinophil-dominant asthma, but the opposite is now considered to be true [36]. In clinical practices, the greatest difficulty encountered during asthma treatment is corticosteroid resistance, which is found in 5 to 10 % of asthma patients. ILC2s are involved in the development of corticosteroid resistance through co-stimulation by IL-33 and thymic stromal lymphopoietin (TSLP), the latter of which strongly activates STAT5 and inhibits apoptosis of ILC2s [32]. While ILC2s exacerbate the severity of symptoms in asthma, amphiregulin produced by ILC2s during influenza virus infection has been shown to be important in the repair of wounded tissue, indicating that ILC2s are involved in the induction of the eosinophilic inflammatory response and tissue repair after inflammation [37] (Fig. 3).

Id2 [16], RORα [38], TCF-1 [39], GATA3 [33, 40, 41, 34], Gfi1 [42], and E4BP4 [43, 44] are transcription factors that are involved in the differentiation of ILC2s, and IL-7 [16] and notch signaling [38] are requisite external factors. While defects in Id2, GATA3, and IL-7 completely impair the differentiation of ILC2s, the absence of RORα, TCF-1, Gfi1, and E4BP4 resulted in a marked reduction in the number of ILC2s. Interestingly, loss of Gfi1 resulted in inhibition of IL-33 but not IL-25-dependent expansion of ILC2s in the lung and MLN [42]. Among the above transcription factors, RORα appears to be the only transcription factor that specifically regulates differentiation of ILC2s but not other ILCs. RORα is highly expressed in ILC2s [16], and its deficiency greatly reduced the number of ILC2s [38, 45]. Interestingly, a small number of ILC2 cells are present in the mesentery of *Rorα* ^*stg*^ mice, a natural mutant mouse lacking functional RORα, and these ILC2 cells produced normal amounts of IL5 and IL-13 in response to IL-33, indicating that RORα is involved in the differentiation of ILC2s but dispensable for cytokine gene expression [34]. In contrast, deletion of *Gata3* in mature ILC2 cells resulted in impaired production of IL-5 and IL-13 without affecting IL-6 production [34]. GATA3 binds to the promoter/enhancer region of *Il5* and *Il13* gens in ILC2 cells, and the deletion of *Cgre* encoding a GATA3-binding site within the *Il13* promoter specifically impaired the expression of IL-13 [34]. GATA3, TCF-1, and Gfi1 but not RORα are important factors for type 2 cytokine production.

The progenitor of ILC2s was first proposed by Yang et al., demonstrating that ILC2s are CLP-derived cells [3]. Fate mapping analysis of *Rag2* expression showed that a fraction of ILC2s once expressed *Rag2*. Subsequently, two types of ILC committed progenitors were described in fetal liver and bone marrow, Lin^−^PLZF^high^IL-7Rα^+^c-Kit^+^α4β7^high^ cells and Lin^−^Id2^+^IL-7Rα^+^CD25^−^α4β7^+^ Flt3^−^cells [7, 4] (Fig. 1). The relationship between these two progenitors has not been elucidated. A Lin^−^Sca-1^hi^Id2^hi^GATA3^hi^ cell (ILC2P) population in the bone marrow was reported to be an ILC2 specific progenitor, but this population has already acquired the ability to produce type 2 cytokines, indicating that the ILC2P population is likely immature ILC2s but not ILC2 progenitors [33]. The theory for the existence of immature ILC2s is backed by data that lack of Gfi1 resulted in the loss of ILC2P but not mature ILC2s in the lung and MLN [42].

There are currently no reports of a relationship between microbiota and ILC2s, and it is known that commensal bacteria are not required for the development of ILC2s since the number of ILC2s does not differ between germ-free and SPF mice [37]. TSLP which accelerates type 2 cytokine production by ILC2s is induced by commensal bacteria in the colon [46], however, suggesting that microbiota may play an important role in the function of ILC2s.

## Group3 ILC

**Fig. 4:**
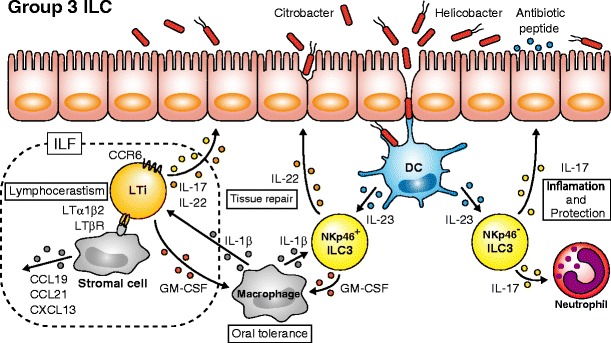
Role of group 3 ILCs. In response to IL-23 produced by dendritic cells (DC), NKp46^−^ILC3s produce IL-17 for the activation of neutrophil migration and protection against bacteria through antibiotic peptide secretion by epithelial cells. NKp46^+^ILC3s produce IL-22 for tissue repair. CCR6^+^ LTi cells in the isolated lymphoid follicles (ILF) interact with stromal cells via lymphotoxin (LT) and LT receptor to enhance chemokine production for lymphocerastism. Macrophages produce IL-1β to induce GM-CSF by both LTi cells and NKp46^+^ILC3s, which seems to be important for the induction of oral tolerance

In 1992, Kelly and Scollay reported on a novel cell subset expressing CD4 but not CD3 in neonatal LN cells [47]. The function of this population was unclear until two research groups identified novel subsets in 1997, which were later called LTi cells: CD4^+^CD3^−^LTβ^+^ cells that express α4β7 and are involved in the formation of lymph nodes [48] and IL-7R^+^ cells that induce formation of Peyer’s patches [49, 48]. During late fetal life, LTi cells produce LTα and LTβ in response to IL-7 or RANKL [50] stimulation and induce VCAM-1 [49, 51] and MAdCAM-1 [52] expression on lymphoid tissue organizer (LTo) cells that will later develop into LN or Peyer’s patch anlagen (Fig. 4). Entry of T cells and B cells into the lymphoid anlagen to complete maturation of lymphoid tissues depends on chemokines such as CXCL13, CCL19, or CCL21 from LTo cells and is a final step of lymphocerastism. Retinoic acid-related orphan receptor γt (RORγt) was demonstrated to be an essential factor for the development of LTi cells, and lack of RORγt resulted in hypoplastic defects of lymph nodes and PP but not spleen, suggesting that LTi cells are involved in the formation of lymph nodes and Peyer’s patches but not spleen [53]. It is currently well-known that LTi cells are not fetal-specific cells but remain in adult tissues, and are involved in the formation of secondary lymphoid tissues such as isolated lymphoid follicles (ILF) in the intestine.

During the middle of a surge in Th17 research in the mid-2000’s, Takatori et al. identified CD4^+^CD3^−^ LTi-like cells, an innate source of IL-17 and IL-22, which express IL-23 receptor, aryl hydrocarbon receptor (AHR) and CCR6, indicating that LTi cells are not only important for the formation of secondary lymphoid tissues but also for host defense by type 3 cytokine production [54]. Around the same time, Satoh-Takayama et al. also reported on a unique population that expresses NKp46 (NCR) in the small intestine [55] and is clearly different from conventional NK cells due to the lack of perforin and limited IFNγ expression. NKp46^+^ cells express RORγt and produce sufficient amounts of IL-22 for protection against *Citrobacter rodentium* infection. A similar subset of cells that produce IL-22 and IL-26 following IL-23 exposure in mucosa-associated lymphoid tissues such as tonsils and Peyer’s patches was characterized in a human study [56]. Collectively, these three reports suggested the presence of a unique population, which is phenotypically similar to LTi cells, but has a different cytokine profile and plays a role in microbial infection (Fig. 4).

LTi cells and IL-22 producing cells are collectively termed group 3 ILC based on the requirement for RORγt for their development. While CCR6^+^ LTi cells are called LTi cells, CCR6^−^ cells are generally termed ILC3s. CCR6 is an important chemokine receptor for the formation of ILFs from cryptopatches by LTi cells after birth. ILC3s can be divided into two subpopulations based on NKp46 expression. NKp46^+^ ILC3, once called ILC22, are exclusively localized in the intestinal lamina propria in the mouse, and a similar population has been reported in human tonsils [57]. NKp46^+^ ILC3s do not produce IL-17 in response to IL-23, but produce IL-22 and function in protection against microbial infection and in tissue homeostasis and repair. On the other hand, NKp46^−^ ILC3, which were previously called ILC17, and LTi cells, produce both IL-17 and IL-22 upon IL-23 stimulation. Furthermore, NKp46^+^ ILC3s require T-bet for their development whereas LTi cells develop independently of T-bet [58]. LTi cells can be divided into two subpopulations based on CD4 expression, but the difference between these subpopulations is not clear because both cells produce similar amounts of IL-22 and IL-17 and express LTα1β2. Similar to cNK cells in group 1 ILC, LTi cells were unique in group 3 ILC, based on their specialized effects on lymphocerastism. As mentioned, identification of a PLZF^high^ precursor clearly demonstrated that NK cells and LTi cells are distinct populations from ILC1, ILC2s, and ILC3s [7]. Although the positional relationship between two ILC progenitors Lin^−^PLZF^high^IL-7Rα^+^c-Kit^+^α4β7^high^ cells termed ILCP and Lin^−^Id2^+^IL-7Rα^+^CD25^−^α4β7^+^ Flt3^−^cells termed CHILP for helper-like ILCs [4] remains unclear, ILCP seem to be a more specific progenitor of the ILCs, because CHILP has been reported to be heterogeneous for expression of PLZF. Moreover, the possibility remains that ILCPs are a composite of the earliest progenitors of ILC1, ILC2s, and ILC3s, based on the result of PLZF^high^ single cell culture. PLZF^high^ cells from fetal liver were co-cultured on OP9 stromal cells for 5–6 days, and development was assessed by flow cytometry analysis. As a result, 7 out of 500 wells developed into all three lineages, while 51 wells developed into double lineage (ILC1,2 : ILC2,3 : ILC3,1 = 9 : 21 : 21 ) and all other wells differentiated into single ILC (ILC1 : ILC2 : ILC3 = 230 : 141 : 71) [7]. It is possible that the PLZF^high^ cell fraction is a mixture of committed progenitors of ILC1, ILC2s, and ILC3s.

There is increasing evidence for crosstalk between commensal microbiota and group 3 ILC. It was controversial whether commensal bacteria are essential for the development of group3 ILC3. While some groups reported the importance of commensal microbiota for the development of NKp46^+^ ILC3 [59, 55], others demonstrated commensal microbiota-independent development of RORγt^+^ ILC3 [60, 61], and Reynders et al. reported normal development and increased IL-22 production of NKp46^+^RORγt^+^ ILC3 in the small intestine of germ-free mice [62]. Recent report has shown that NKp46 expression is downstream of T-bet that is induced by microbiota and thus NKp46^+^T-bet^+^ ILC3s are mostly dependent on microbiota [63]. In addition, CCR6^−^ ILC3s are dependent upon the microbiota, while TLi cells are largely independent of the microbiota [63]. Because it is difficult to discern whether environmental factors including commensal bacteria are involved in the differentiation, mechanisms how microbiota regulates the homeostasis of ILC3s remain to be elucidated.

Infection with *Salmonella* and *Pseudomonas* species induces IL-1β production by intestinal mononuclear phagocytes through the NLRC4 inflammasome pathway, which is critical for the upregulation of endothelial adhesion molecules and neutrophil recruitment in the intestine [64]. Recently, Mortha et al. reported that ILC3s in the isolated lymphoid follicles produce GM-CSF to promote oral tolerance in response to IL-1β from intestinal macrophages [65]. They demonstrated that LTi cells and NKp46^+^ ILC3s but not NKp46^+^RORγt^−^ NK cells produce GM-CSF in steady-state conditions. GM-CSF production was absent in newborn mice, and strongly reduced by treatment with broad-spectrum antibiotics in both the small and large intestine in adult mice, suggesting that commensal bacteria are essential for this production (Fig. 4). ILC3s also play a role in epithelial cell fucosylation, which is important for the integrity of epithelial cells and protection against bacterial infection [66], indicating that both ILC2s and ILC3s are important in the maintenance of epithelial integrity.

AHR seems to play a role in ILC3 differentiation and function. *Ahr*-deficient mice exhibited normal development of CCR6^+^ LTi cells but reduced numbers of CCR6^−^ ILC3 cells [67, 61, 63, 68]. Consistent with these observations, lymph nodes and Peyer’s patches that are formed during fetal stages are mostly normal in *Ahr*-deficient mice, whereas cryptopatches and ILF are absent in the absence of *Ahr* expression. Although the mechanisms by which AHR controls ILC3 differentiation are unknown, AHR may regulate genes involved in the differentiation of ILC3. For example, AHR is known to bind to the promoter of the *c-Kit* gene [69]. AHR is also known to act as a sensor of environmental cues by sensing various small molecules. AHR binds nutrient-derived glucobrassicins that are contained in vegetables of the *Brassicaceae* family (for example, Broccoli) and drives postnatal expansion of CCR6^−^ ILC3 and intraepithelial γδ T cells [69, 70]. It is thus possible that phytochemicals control the development and maintenance of the immune system at the intestinal barrier surfaces.

In addition, recent studies have shown the importance of another nutrient, vitamin A, in the differentiation of ILC3s. *Rarα*-deficient mice lacking retinoic acid receptor (RAR) α in hematopoietic cells have a reduced number of fetal LTi cell partly because RARα binds the promoter region of the *Rorc* gene and upregulates the expression of RORγt. As a result, adult *Rara*-deficient mice have smaller lymphoid organs and are less tolerant to viral infections [71]. Spencer et al. (2014) reported that deprivation of vitamin A in adult mice resulted in the reduction of IL-22-producing ILC3 and impaired immunity against *C. rodentium* [72]. Intriguingly, the number of ILC2s increased in these mice, demonstrating that the mechanisms balancing ILC2 and ILC3 in mucosal barrier surfaces are nutrient-dependent.

## Conclusion and perspectives

We have discussed the differentiation and function of ILCs and their relationship between environmental factors including commensal microbiota. As discussed, ILCs are distributed throughout the epithelial compartment and are involved in the maintenance of epithelial integrity and protection against various invading microbes. Commensal microbiota likely play important roles in the maintenance of epithelial integrity through their effects on ILC differentiation and function. It is well known that environmental factors including commensal microbiota affect cellular characteristics through epigenetic modifications. Therefore, it is of interest to consider evidence for the possible involvement of commensal microbiota in the plasticity between distinct ILCs. IFN-γ-producing ILC1 that express the receptor for IL-7 and lack NK cell markers and lytic enzymes, arise from the conversion of RORγt^+^ ILC3s under the influence of IL-12 during inflammation [11]. Similarly, RORγt^+^ IFN-γ-producing cells can be induced from human ILC3s in vitro by culturing with IL-2 [73–75]. In mice, fate-mapping experiments showed that some IFN-γ-producing ILC1 cells are indeed derived from RORγt^+^ ILC3s [4, 76]. These studies collectively suggest that a certain fraction of ILC1 might stem from ILC3s. Compared to ILC1 and ILC3s, ILC2s seem to be a relatively stable subset. However, it is still possible that there is plasticity between ILC2s and other ILC subsets [42, 77]. Crellin et al. reported that human ILC3s can produce IL-5 and IL-13 when stimulated with TLR2 ligands [77]. In addition, Spooner et al. showed that lack of the Gfi1 gene resulted in the loss of GATA-3 expression and coexpression of IL-13 and IL-17 [42]. They showed that Gfi1 suppresses the phenotypic characteristics of ILC3s through suppression of the *Sox4-Rorc* axis that is required for the expression of the *Il17a* gene [42]. The mechanisms for the stabilization of the characteristics of ILCs and those driving the plasticity between distinct ILCs and involvement of commensal microbiota remain to be elucidated in future studies.

## References

[1] Chen H, Ray-Gallet D, Zhang P, Hetherington CJ, Gonzalez DA, Zhang DE, Moreau-Gachelin F, Tenen DG (1995). PU.1 (Spi-1) autoregulates its expression in myeloid cells. Oncogene.

[2] Spits H, Artis D, Colonna M, Diefenbach A, Di Santo JP, Eberl G, Koyasu S, Locksley RM, McKenzie AN, Mebius RE, Powrie F, Vivier E (2013). Innate lymphoid cells—a proposal for uniform nomenclature. Nat Rev Immunol.

[3] Yang Q, Saenz SA, Zlotoff DA, Artis D, Bhandoola A (2011). Cutting edge: natural helper cells derive from lymphoid progenitors. J Immunol (Baltimore, Md : 1950).

[4] Klose CS, Flach M, Mohle L, Rogell L, Hoyler T, Ebert K, Fabiunke C, Pfeifer D, Sexl V, Fonseca-Pereira D, Domingues RG, Veiga-Fernandes H, Arnold SJ, Busslinger M, Dunay IR, Tanriver Y, Diefenbach A (2014). Differentiation of type 1 ILCs from a common progenitor to all helper-like innate lymphoid cell lineages. Cell.

[5] Savage AK, Constantinides MG, Han J, Picard D, Martin E, Li B, Lantz O, Bendelac A (2008). The transcription factor PLZF directs the effector program of the NKT cell lineage. Immunity.

[6] Kovalovsky D, Uche OU, Eladad S, Hobbs RM, Yi W, Alonzo E, Chua K, Eidson M, Kim HJ, Im JS, Pandolfi PP, Sant'Angelo DB (2008). The BTB-zinc finger transcriptional regulator PLZF controls the development of invariant natural killer T cell effector functions. Nat Immunol.

[7] Constantinides MG, McDonald BD, Verhoef PA, Bendelac A (2014). A committed precursor to innate lymphoid cells. Nature.

[8] Kiessling R, Klein E, Wigzell H (1975). “Natural” killer cells in the mouse. I. Cytotoxic cells with specificity for mouse Moloney leukemia cells. Specificity and distribution according to genotype. Eur J Immunol.

[9] Kiessling R, Klein E, Pross H, Wigzell H (1975). “Natural” killer cells in the mouse. II. Cytotoxic cells with specificity for mouse Moloney leukemia cells. Characteristics of the killer cell. Eur J Immunol.

[10] Vosshenrich CA, Garcia-Ojeda ME, Samson-Villeger SI, Pasqualetto V, Enault L, Richard-Le Goff O, Corcuff E, Guy-Grand D, Rocha B, Cumano A, Rogge L, Ezine S, Di Santo JP (2006). A thymic pathway of mouse natural killer cell development characterized by expression of GATA-3 and CD127. Nat Immunol.

[11] Bernink JH, Peters CP, Munneke M, te Velde AA, Meijer SL, Weijer K, Hreggvidsdottir HS, Heinsbroek SE, Legrand N, Buskens CJ, Bemelman WA, Mjosberg JM, Spits H (2013). Human type 1 innate lymphoid cells accumulate in inflamed mucosal tissues. Nat Immunol.

[12] Fuchs A, Vermi W, Lee JS, Lonardi S, Gilfillan S, Newberry RD, Cella M, Colonna M (2013). Intraepithelial type 1 innate lymphoid cells are a unique subset of IL-12- and IL-15-responsive IFN-gamma-producing cells. Immunity.

[13] He YW, Malek TR (1996). Interleukin-7 receptor alpha is essential for the development of gamma delta + T cells, but not natural killer cells. J Exp Med.

[14] Carotta S, Pang SH, Nutt SL, Belz GT (2011). Identification of the earliest NK-cell precursor in the mouse BM. Blood.

[15] Ganal SC, Sanos SL, Kallfass C, Oberle K, Johner C, Kirschning C, Lienenklaus S, Weiss S, Staeheli P, Aichele P, Diefenbach A (2012). Priming of natural killer cells by nonmucosal mononuclear phagocytes requires instructive signals from commensal microbiota. Immunity.

[16] Moro K, Yamada T, Tanabe M, Takeuchi T, Ikawa T, Kawamoto H, Furusawa J, Ohtani M, Fujii H, Koyasu S (2010). Innate production of T(H)2 cytokines by adipose tissue-associated c-Kit(+)Sca-1(+) lymphoid cells. Nature.

[17] Fort MM, Cheung J, Yen D, Li J, Zurawski SM, Lo S, Menon S, Clifford T, Hunte B, Lesley R, Muchamuel T, Hurst SD, Zurawski G, Leach MW, Gorman DM, Rennick DM (2001). IL-25 induces IL-4, IL-5, and IL-13 and Th2-associated pathologies in vivo. Immunity.

[18] Schmitz J, Owyang A, Oldham E, Song Y, Murphy E, McClanahan TK, Zurawski G, Moshrefi M, Qin J, Li X, Gorman DM, Bazan JF, Kastelein RA (2005). IL-33, an interleukin-1-like cytokine that signals via the IL-1 receptor-related protein ST2 and induces T helper type 2-associated cytokines. Immunity.

[19] Neill DR, Wong SH, Bellosi A, Flynn RJ, Daly M, Langford TK, Bucks C, Kane CM, Fallon PG, Pannell R, Jolin HE, McKenzie AN (2010). Nuocytes represent a new innate effector leukocyte that mediates type-2 immunity. Nature.

[20] Chang YJ, Kim HY, Albacker LA, Baumgarth N, McKenzie AN, Smith DE, Dekruyff RH, Umetsu DT (2011). Innate lymphoid cells mediate influenza-induced airway hyper-reactivity independently of adaptive immunity. Nat Immunol.

[21] Ikutani M, Yanagibashi T, Ogasawara M, Tsuneyama K, Yamamoto S, Hattori Y, Kouro T, Itakura A, Nagai Y, Takaki S, Takatsu K (2012). Identification of innate IL-5-producing cells and their role in lung eosinophil regulation and antitumor immunity. J Immunol (Baltimore, Md : 1950).

[22] Price AE, Liang HE, Sullivan BM, Reinhardt RL, Eisley CJ, Erle DJ, Locksley RM (2010). Systemically dispersed innate IL-13-expressing cells in type 2 immunity. Proc Natl Acad Sci U S A.

[23] Roediger B, Kyle R, Yip KH, Sumaria N, Guy TV, Kim BS, Mitchell AJ, Tay SS, Jain R, Forbes-Blom E, Chen X, Tong PL, Bolton HA, Artis D, Paul WE, de St F, Groth B, Grimbaldeston MA, Le Gros G, Weninger W (2013). Cutaneous immunosurveillance and regulation of inflammation by group 2 innate lymphoid cells. Nat Immunol.

[24] Imai Y, Yasuda K, Sakaguchi Y, Haneda T, Mizutani H, Yoshimoto T, Nakanishi K, Yamanishi K (2013). Skin-specific expression of IL-33 activates group 2 innate lymphoid cells and elicits atopic dermatitis-like inflammation in mice. Proc Natl Acad Sci U S A.

[25] Hardman CS, Panova V, McKenzie AN (2013). IL-33 citrine reporter mice reveal the temporal and spatial expression of IL-33 during allergic lung inflammation. Eur J Immunol.

[26] Hung LY, Lewkowich IP, Dawson LA, Downey J, Yang Y, Smith DE, Herbert DR (2013). IL-33 drives biphasic IL-13 production for noncanonical type 2 immunity against hookworms. Proc Natl Acad Sci U S A.

[27] Klein Wolterink RG, Kleinjan A, van Nimwegen M, Bergen I, de Bruijn M, Levani Y, Hendriks RW (2012). Pulmonary innate lymphoid cells are major producers of IL-5 and IL-13 in murine models of allergic asthma. Eur J Immunol.

[28] Licona-Limon P, Kim LK, Palm NW, Flavell RA (2013). TH2, allergy and group 2 innate lymphoid cells. Nat Immunol.

[29] McHedlidze T, Waldner M, Zopf S, Walker J, Rankin AL, Schuchmann M, Voehringer D, McKenzie AN, Neurath MF, Pflanz S, Wirtz S (2013). Interleukin-33-dependent innate lymphoid cells mediate hepatic fibrosis. Immunity.

[30] Molofsky AB, Nussbaum JC, Liang HE, Van Dyken SJ, Cheng LE, Mohapatra A, Chawla A, Locksley RM (2013). Innate lymphoid type 2 cells sustain visceral adipose tissue eosinophils and alternatively activated macrophages. J Exp Med.

[31] Salimi M, Barlow JL, Saunders SP, Xue L, Gutowska-Owsiak D, Wang X, Huang LC, Johnson D, Scanlon ST, McKenzie AN, Fallon PG, Ogg GS (2013). A role for IL-25 and IL-33-driven type-2 innate lymphoid cells in atopic dermatitis. J Exp Med.

[32] Kabata H, Moro K, Fukunaga K, Suzuki Y, Miyata J, Masaki K, Betsuyaku T, Koyasu S, Asano K (2013). Thymic stromal lymphopoietin induces corticosteroid resistance in natural helper cells during airway inflammation. Nat Commun.

[33] Hoyler T, Klose CS, Souabni A, Turqueti-Neves A, Pfeifer D, Rawlins EL, Voehringer D, Busslinger M, Diefenbach A (2012). The transcription factor GATA-3 controls cell fate and maintenance of type 2 innate lymphoid cells. Immunity.

[34] Furusawa J, Moro K, Motomura Y, Okamoto K, Zhu J, Takayanagi H, Kubo M, Koyasu S (2013). Critical role of p38 and GATA3 in natural helper cell function. J Immunol (Baltimore, Md : 1950).

[35] Halim TY, Krauss RH, Sun AC, Takei F (2012). Lung natural helper cells are a critical source of Th2 cell-type cytokines in protease allergen-induced airway inflammation. Immunity.

[36] Holgate ST, Polosa R (2006). The mechanisms, diagnosis, and management of severe asthma in adults. Lancet.

[37] Monticelli LA, Sonnenberg GF, Abt MC, Alenghat T, Ziegler CG, Doering TA, Angelosanto JM, Laidlaw BJ, Yang CY, Sathaliyawala T, Kubota M, Turner D, Diamond JM, Goldrath AW, Farber DL, Collman RG, Wherry EJ, Artis D (2011). Innate lymphoid cells promote lung-tissue homeostasis after infection with influenza virus. Nat Immunol.

[38] Wong SH, Walker JA, Jolin HE, Drynan LF, Hams E, Camelo A, Barlow JL, Neill DR, Panova V, Koch U, Radtke F, Hardman CS, Hwang YY, Fallon PG, McKenzie AN (2012). Transcription factor RORalpha is critical for nuocyte development. Nat Immunol.

[39] Yang Q, Monticelli LA, Saenz SA, Chi AW, Sonnenberg GF, Tang J, De Obaldia ME, Bailis W, Bryson JL, Toscano K, Huang J, Haczku A, Pear WS, Artis D, Bhandoola A (2013). T cell factor 1 is required for group 2 innate lymphoid cell generation. Immunity.

[40] Klein Wolterink RG, Serafini N, van Nimwegen M, Vosshenrich CA, de Bruijn MJ, Fonseca Pereira D, Veiga Fernandes H, Hendriks RW, Di Santo JP (2013). Essential, dose-dependent role for the transcription factor Gata3 in the development of IL-5+ and IL-13+ type 2 innate lymphoid cells. Proc Natl Acad Sci U S A.

[41] Mjosberg J, Bernink J, Golebski K, Karrich JJ, Peters CP, Blom B, te Velde AA, Fokkens WJ, van Drunen CM, Spits H (2012). The transcription factor GATA3 is essential for the function of human type 2 innate lymphoid cells. Immunity.

[42] Spooner CJ, Lesch J, Yan D, Khan AA, Abbas A, Ramirez-Carrozzi V, Zhou M, Soriano R, Eastham-Anderson J, Diehl L, Lee WP, Modrusan Z, Pappu R, Xu M, DeVoss J, Singh H (2013). Specification of type 2 innate lymphocytes by the transcriptional determinant Gfi1. Nat Immunol.

[43] Geiger TL, Abt MC, Gasteiger G, Firth MA, O'Connor MH, Geary CD, O'Sullivan TE, van den Brink MR, Pamer EG, Hanash AM, Sun JC (2014). Nfil3 is crucial for development of innate lymphoid cells and host protection against intestinal pathogens. J Exp Med.

[44] Seillet C, Rankin LC, Groom JR, Mielke LA, Tellier J, Chopin M, Huntington ND, Belz GT, Carotta S (2014). Nfil3 is required for the development of all innate lymphoid cell subsets. J Exp Med.

[45] Halim TY, MacLaren A, Romanish MT, Gold MJ, McNagny KM, Takei F (2012). Retinoic-acid-receptor-related orphan nuclear receptor alpha is required for natural helper cell development and allergic inflammation. Immunity.

[46] Mosconi I, Geuking MB, Zaiss MM, Massacand JC, Aschwanden C, Kwong Chung CK, McCoy KD, Harris NL (2013). Intestinal bacteria induce TSLP to promote mutualistic T-cell responses. Mucosal Immunol.

[47] Kelly KA, Scollay R (1992). Seeding of neonatal lymph nodes by T cells and identification of a novel population of CD3-CD4+ cells. Eur J Immunol.

[48] Mebius RE, Rennert P, Weissman IL (1997). Developing lymph nodes collect CD4 + CD3- LTbeta + cells that can differentiate to APC, NK cells, and follicular cells but not T or B cells. Immunity.

[49] Adachi S, Yoshida H, Kataoka H, Nishikawa S (1997). Three distinctive steps in Peyer's patch formation of murine embryo. Int Immunol.

[50] Kong YY, Yoshida H, Sarosi I, Tan HL, Timms E, Capparelli C, Morony S, Oliveira-dos-Santos AJ, Van G, Itie A, Khoo W, Wakeham A, Dunstan CR, Lacey DL, Mak TW, Boyle WJ, Penninger JM (1999). OPGL is a key regulator of osteoclastogenesis, lymphocyte development and lymph-node organogenesis. Nature.

[51] Adachi S, Yoshida H, Honda K, Maki K, Saijo K, Ikuta K, Saito T, Nishikawa SI (1998). Essential role of IL-7 receptor alpha in the formation of Peyer's patch anlage. Int Immunol.

[52] Eberl G, Littman DR (2003). The role of the nuclear hormone receptor RORgammat in the development of lymph nodes and Peyer's patches. Immunol Rev.

[53] Sun Z, Unutmaz D, Zou YR, Sunshine MJ, Pierani A, Brenner-Morton S, Mebius RE, Littman DR (2000). Requirement for RORgamma in thymocyte survival and lymphoid organ development. Science.

[54] Takatori H, Kanno Y, Watford WT, Tato CM, Weiss G, Ivanov II, Littman DR, O'Shea JJ (2009). Lymphoid tissue inducer-like cells are an innate source of IL-17 and IL-22. J Exp Med.

[55] Satoh-Takayama N, Vosshenrich CA, Lesjean-Pottier S, Sawa S, Lochner M, Rattis F, Mention JJ, Thiam K, Cerf-Bensussan N, Mandelboim O, Eberl G, Di Santo JP (2008). Microbial flora drives interleukin 22 production in intestinal NKp46+ cells that provide innate mucosal immune defense. Immunity.

[56] Cella M, Fuchs A, Vermi W, Facchetti F, Otero K, Lennerz JK, Doherty JM, Mills JC, Colonna M (2009). A human natural killer cell subset provides an innate source of IL-22 for mucosal immunity. Nature.

[57] Cupedo T, Crellin NK, Papazian N, Rombouts EJ, Weijer K, Grogan JL, Fibbe WE, Cornelissen JJ, Spits H (2009). Human fetal lymphoid tissue-inducer cells are interleukin 17-producing precursors to RORC + CD127+ natural killer-like cells. Nat Immunol.

[58] Rankin LC, Groom JR, Chopin M, Herold MJ, Walker JA, Mielke LA, McKenzie AN, Carotta S, Nutt SL, Belz GT (2013). The transcription factor T-bet is essential for the development of NKp46+ innate lymphocytes via the Notch pathway. Nat Immunol.

[59] Sanos SL, Bui VL, Mortha A, Oberle K, Heners C, Johner C, Diefenbach A (2009). RORgammat and commensal microflora are required for the differentiation of mucosal interleukin 22-producing NKp46+ cells. Nat Immunol.

[60] Sawa S, Cherrier M, Lochner M, Satoh-Takayama N, Fehling HJ, Langa F, Di Santo JP, Eberl G (2010). Lineage relationship analysis of RORgammat + innate lymphoid cells. Science.

[61] Lee JS, Cella M, McDonald KG, Garlanda C, Kennedy GD, Nukaya M, Mantovani A, Kopan R, Bradfield CA, Newberry RD, Colonna M (2012). AHR drives the development of gut ILC22 cells and postnatal lymphoid tissues via pathways dependent on and independent of Notch. Nat Immunol.

[62] Reynders A, Yessaad N, Vu Manh TP, Dalod M, Fenis A, Aubry C, Nikitas G, Escaliere B, Renauld JC, Dussurget O, Cossart P, Lecuit M, Vivier E, Tomasello E (2011). Identity, regulation and in vivo function of gut NKp46 + RORgammat + and NKp46 + RORgammat- lymphoid cells. EMBO J.

[63] Klose CS, Kiss EA, Schwierzeck V, Ebert K, Hoyler T, d’Hargues Y, Goppert N, Croxford AL, Waisman A, Tanriver Y, Diefenbach A (2013). A T-bet gradient controls the fate and function of CCR6-RORgammat + innate lymphoid cells. Nature.

[64] Franchi L, Kamada N, Nakamura Y, Burberry A, Kuffa P, Suzuki S, Shaw MH, Kim YG, Nunez G (2012). NLRC4-driven production of IL-1beta discriminates between pathogenic and commensal bacteria and promotes host intestinal defense. Nat Immunol.

[65] Mortha A, Chudnovskiy A, Hashimoto D, Bogunovic M, Spencer SP, Belkaid Y, Merad M (2014). Microbiota-dependent crosstalk between macrophages and ILC3 promotes intestinal homeostasis. Science.

[66] Goto Y, Obata T, Kunisawa J, Sato S, Ivanov II, Lamichhane A, Takeyama N, Kamioka M, Sakamoto M, Matsuki T, Setoyama H, Imaoka A, Uematsu S, Akira S, Domino SE, Kulig P, Becher B, Renauld JC, Sasakawa C, Umesaki Y, Benno Y, Kiyono H (2014). Innate lymphoid cells regulate intestinal epithelial cell glycosylation. Science.

[67] Kiss EA, Vonarbourg C, Kopfmann S, Hobeika E, Finke D, Esser C, Diefenbach A (2011). Natural aryl hydrocarbon receptor ligands control organogenesis of intestinal lymphoid follicles. Science.

[68] Qiu J, Heller JJ, Guo X, Chen ZM, Fish K, Fu YX, Zhou L (2012). The aryl hydrocarbon receptor regulates gut immunity through modulation of innate lymphoid cells. Immunity.

[69] Kiss EA, Vonarbourg C (2012). Aryl hydrocarbon receptor: a molecular link between postnatal lymphoid follicle formation and diet. Gut Microbes.

[70] Li Y, Innocentin S, Withers DR, Roberts NA, Gallagher AR, Grigorieva EF, Wilhelm C, Veldhoen M (2011). Exogenous stimuli maintain intraepithelial lymphocytes via aryl hydrocarbon receptor activation. Cell.

[71] van de Pavert SA, Ferreira M, Domingues RG, Ribeiro H, Molenaar R, Moreira-Santos L, Almeida FF, Ibiza S, Barbosa I, Goverse G, Labao-Almeida C, Godinho-Silva C, Konijn T, Schooneman D, O'Toole T, Mizee MR, Habani Y, Haak E, Santori FR, Littman DR, Schulte-Merker S, Dzierzak E, Simas JP, Mebius RE, Veiga-Fernandes H (2014). Maternal retinoids control type 3 innate lymphoid cells and set the offspring immunity. Nature.

[72] Spencer SP, Wilhelm C, Yang Q, Hall JA, Bouladoux N, Boyd A, Nutman TB, Urban JF, Wang J, Ramalingam TR, Bhandoola A, Wynn TA, Belkaid Y (2014). Adaptation of innate lymphoid cells to a micronutrient deficiency promotes type 2 barrier immunity. Science.

[73] Cella M, Otero K, Colonna M (2010). Expansion of human NK-22 cells with IL-7, IL-2, and IL-1beta reveals intrinsic functional plasticity. Proc Natl Acad Sci U S A.

[74] Hughes T, Becknell B, McClory S, Briercheck E, Freud AG, Zhang X, Mao H, Nuovo G, Yu J, Caligiuri MA (2009). Stage 3 immature human natural killer cells found in secondary lymphoid tissue constitutively and selectively express the TH 17 cytokine interleukin-22. Blood.

[75] Hughes T, Becknell B, Freud AG, McClory S, Briercheck E, Yu J, Mao C, Giovenzana C, Nuovo G, Wei L, Zhang X, Gavrilin MA, Wewers MD, Caligiuri MA (2010). Interleukin-1beta selectively expands and sustains interleukin-22+ immature human natural killer cells in secondary lymphoid tissue. Immunity.

[76] Vonarbourg C, Mortha A, Bui VL, Hernandez PP, Kiss EA, Hoyler T, Flach M, Bengsch B, Thimme R, Holscher C, Honig M, Pannicke U, Schwarz K, Ware CF, Finke D, Diefenbach A (2010). Regulated expression of nuclear receptor RORgammat confers distinct functional fates to NK cell receptor-expressing RORgammat(+) innate lymphocytes. Immunity.

[77] Crellin NK, Trifari S, Kaplan CD, Satoh-Takayama N, Di Santo JP, Spits H (2010). Regulation of cytokine secretion in human CD127(+) LTi-like innate lymphoid cells by Toll-like receptor 2. Immunity.

